# Transformation to Small‐Cell Lung Carcinoma and Then to Sarcomatoid Carcinoma in an EGFR‐Mutant Lung Adenocarcinoma Patient During Systemic Therapy: A Case Report

**DOI:** 10.1111/1759-7714.70397

**Published:** 2026-11-03

**Authors:** Bingyu Huang, Tao Lu, Ruping Hong, Xiaoyan Liu, Xiaoxing Gao, Yan Xu, Minjiang Chen, Wei Zhong, Mengzhao Wang, Jing Zhao

**Affiliations:** ^1^ Department of Internal Medicine Peking Union Medical College Hospital, Chinese Academy of Medical Sciences & Peking Union Medical College Beijing China; ^2^ Department of Pathology Peking Union Medical College Hospital, Chinese Academy of Medical Sciences & Peking Union Medical College Beijing China; ^3^ Department of Pulmonary and Critical Care Medicine Peking Union Medical College Hospital, Chinese Academy of Medical Sciences & Peking Union Medical College Beijing China

**Keywords:** EGFR mutation, histological transformation, lung adenocarcinoma, sarcomatoid carcinoma, small‐cell lung carcinoma

## Abstract

This case exemplifies stepwise histological transformations of an initially EGFR‐mutant lung adenocarcinoma driven by tumor lineage plasticity under therapeutic pressure. Initial resistance to osimertinib manifested as transformation to combined small‐cell lung carcinoma, associated with concurrent TP53/RB1 co‐inactivation and the acquisition of an epidermal growth factor receptor (EGFR) C797S mutation. Subsequent progression after chemotherapy featured a rare sarcomatoid transformation, with the tumor retaining the original EGFR Exon 19 deletion, supporting clonal evolution. This phenotypic shift, indicative of epithelial‐to‐mesenchymal transition (EMT), may have been associated with PTEN loss and potential activation of the PI3K/AKT/mTOR pathway. The serial emergence of neuroendocrine and sarcomatoid phenotypes underscores the critical need for rebiopsy to guide therapy and highlights the profound adaptability of EGFR‐mutant tumors, which remain a major therapeutic challenge.

## Graphical abstract

An EGFR‐mutant lung adenocarcinoma underwent sequential histological transformations to combined small‐cell carcinoma and sarcomatoid carcinoma during systemic therapy. Serial re‐biopsy demonstrated dynamic tumor evolution and molecular alterations associated with treatment resistance.

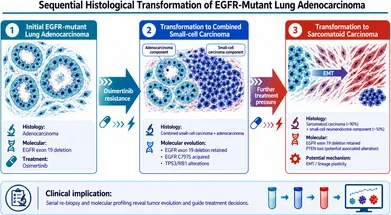

## 1 Case Presentation

A 41‐year‐old male was diagnosed with metastatic EGFR‐mutant lung adenocarcinoma harboring an EGFR Exon 19 deletion (c.2237_2255delinsT, p.E746_S752delinsV) (Figure 1) and concurrent TP53/RB1 alterations. The initial biopsy demonstrated adenocarcinoma morphology, with immunohistochemical staining positive for CK7 and TTF‐1 and negative for p40 and ALK‐D5F3. PD‐L1 expression was low (tumor proportion score [TPS] < 1%), and no morphological evidence of neuroendocrine differentiation was identified. Osimertinib was administered as first‐line therapy. After 16 months, the original lesion in the right lower lung enlarged, and a new nodule appeared in the right middle lobe. Second‐line therapy with pemetrexed and carboplatin plus pembrolizumab was initiated. After two cycles, chest computed tomography (CT) scan demonstrated progression of the pulmonary lesions. Serum tumor markers were elevated, with pro‐gastrin‐releasing peptide (ProGRP) at 83.4 pg/mL, and neuron specific enolase (NSE) at 21.4 ng/mL. A rebiopsy of the primary lesion was subsequently performed. Immunohistochemical staining of the biopsy specimen showed positivity for CD56 (partial+), CgA (focal+), and Syn (partial+), with a Ki‐67 labeling index of 95%, supporting the diagnosis of combined small‐cell carcinoma (c‐SCLC) (Figure 2). Histological examination of the same biopsy specimen revealed two distinct components, consisting of approximately 60% small‐cell carcinoma and 40% adenocarcinoma, indicating substantial intratumoral heterogeneity. Next‐generation sequencing (NGS) identified persistence of the EGFR Exon 19 deletion, alongside inactivating mutations in TP53 and RB1, as well as a newly acquired EGFR Exon 20 C797S mutation (Figure 3). Given the coexistence of small‐cell carcinoma and adenocarcinoma components, treatment was subsequently switched to nab‐paclitaxel and carboplatin plus pembrolizumab to provide therapeutic coverage for both histological components. The primary lesion remained stable after six cycles of this regimen, followed by two cycles of pembrolizumab maintenance therapy alone. Approximately 6 months later, the patient presented with hemoptysis and headache. Chest CT scan showed significant enlargement of the primary lesion and mediastinal lymph nodes. Magnetic resonance imaging revealed intracranial metastases. Given the rapid progression and predominant small‐cell carcinoma phenotype, therapy with etoposide plus carboplatin was initiated. However, chest CT scan after the first cycle indicated progressive disease. Topotecan was then administered concurrently with whole‐brain radiotherapy. After one cycle of topotecan, the pulmonary lesions continued to progress rapidly. Rigid bronchoscopy was performed, revealing a neoplasm obstructing the right main bronchus. Biopsy of the lesion revealed a heterogeneous tumor predominantly composed of spindle‐shaped cells, with a minor component forming small‐cell nests. Based on the morphological features and immunohistochemical profile, the tumor was characterized as sarcomatoid carcinoma with a minor small‐cell neuroendocrine carcinoma component (approximately 90% and 10%, respectively). The small‐cell component showed positivity for TTF‐1, chromogranin A (CgA), and AE1/AE3, whereas the spindle‐cell component exhibited only focal weak AE1/AE3 expression. Additional immunohistochemical findings included partial p63 positivity and partial Vimentin positivity (Figure 4). NGS demonstrated persistence of the original EGFR exon 19 deletion, further supporting a common clonal origin. The patient subsequently received one cycle of gemcitabine and two cycles of the antibody‐drug conjugate sacituzumab tirumotecan; however, the pulmonary lesions continued to progress. The patient is currently receiving docetaxel therapy. Detailed radiological change is presented in Figure 5.

**FIGURE 1:**
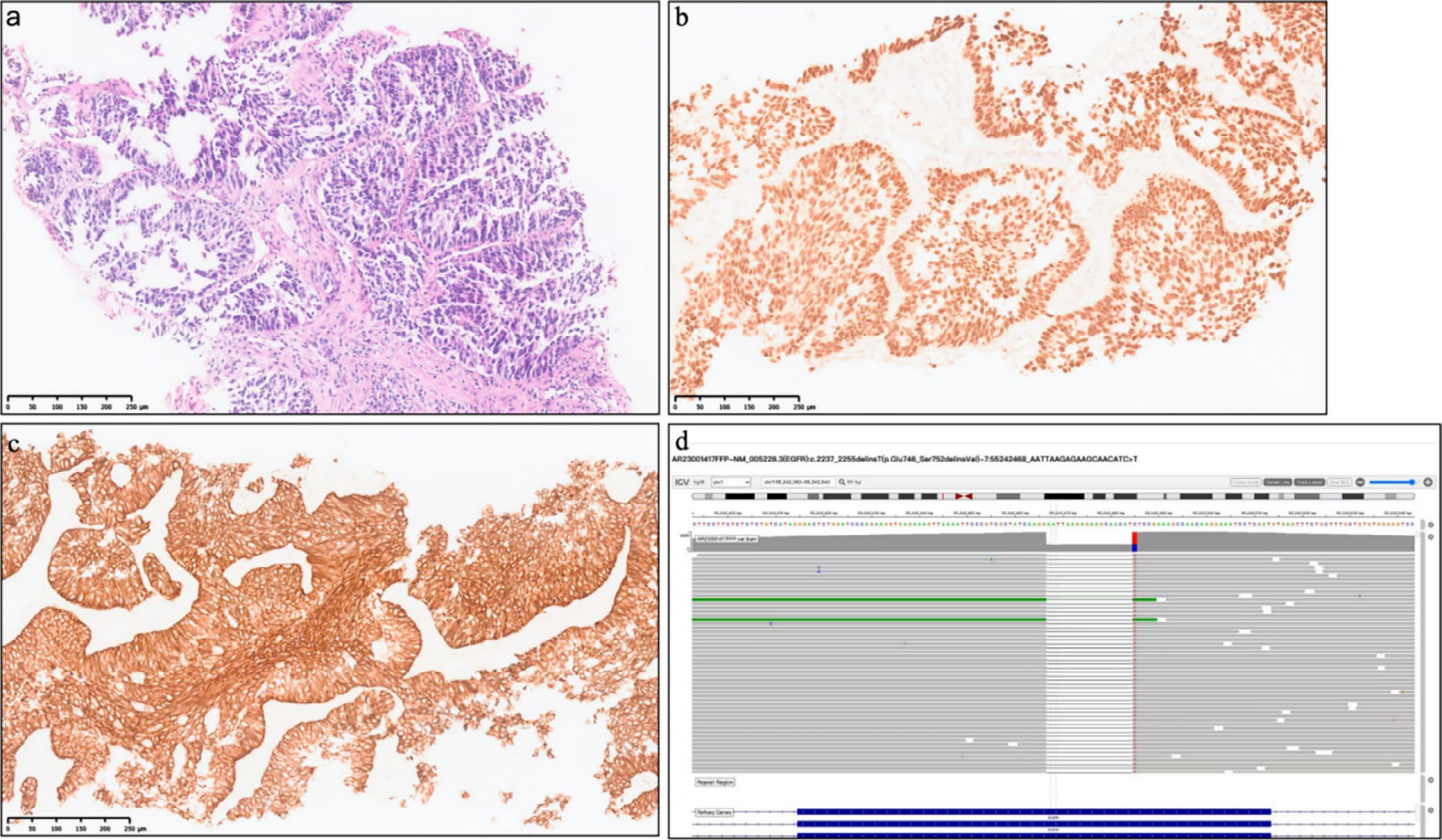
First biopsy specimen showing lung adenocarcinoma. (a) Hematoxylin and eosin (H&E) staining showing adenocarcinoma morphology. (b, c) Immunohistochemical staining for thyroid transcription factor‐1 (TTF‐1) and cytokeratin 7 (CK7). (d) NGS showing an EGFR Exon 19 deletion.

**FIGURE 2:**
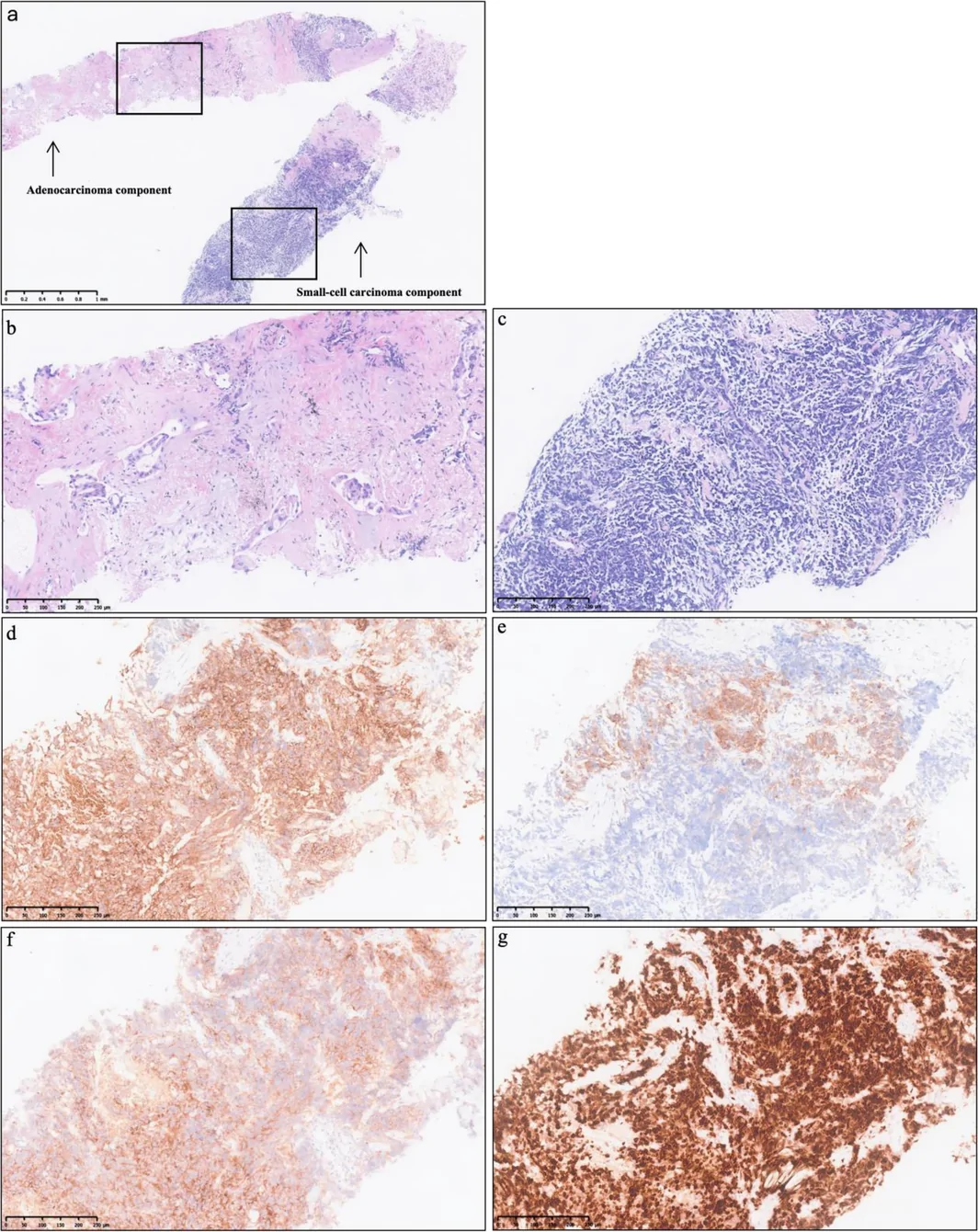
Histological and immunohistochemical features of the second biopsy specimen showing combined small‐cell carcinoma (c‐SCLC). (a) H&E staining showing adenocarcinoma and small‐cell carcinoma components within the same biopsy specimen. Enlarged views of the adenocarcinoma component and small‐cell carcinoma component are shown in (b) and (c). (d–g) Immunohistochemical staining highlighting neuroendocrine differentiation in the small‐cell carcinoma component, including (d) cluster of differentiation 56 (CD56), (e) chromogranin A (CgA), (f) synaptophysin (Syn), and (g) Ki‐67 labeling index.

**FIGURE 3:**
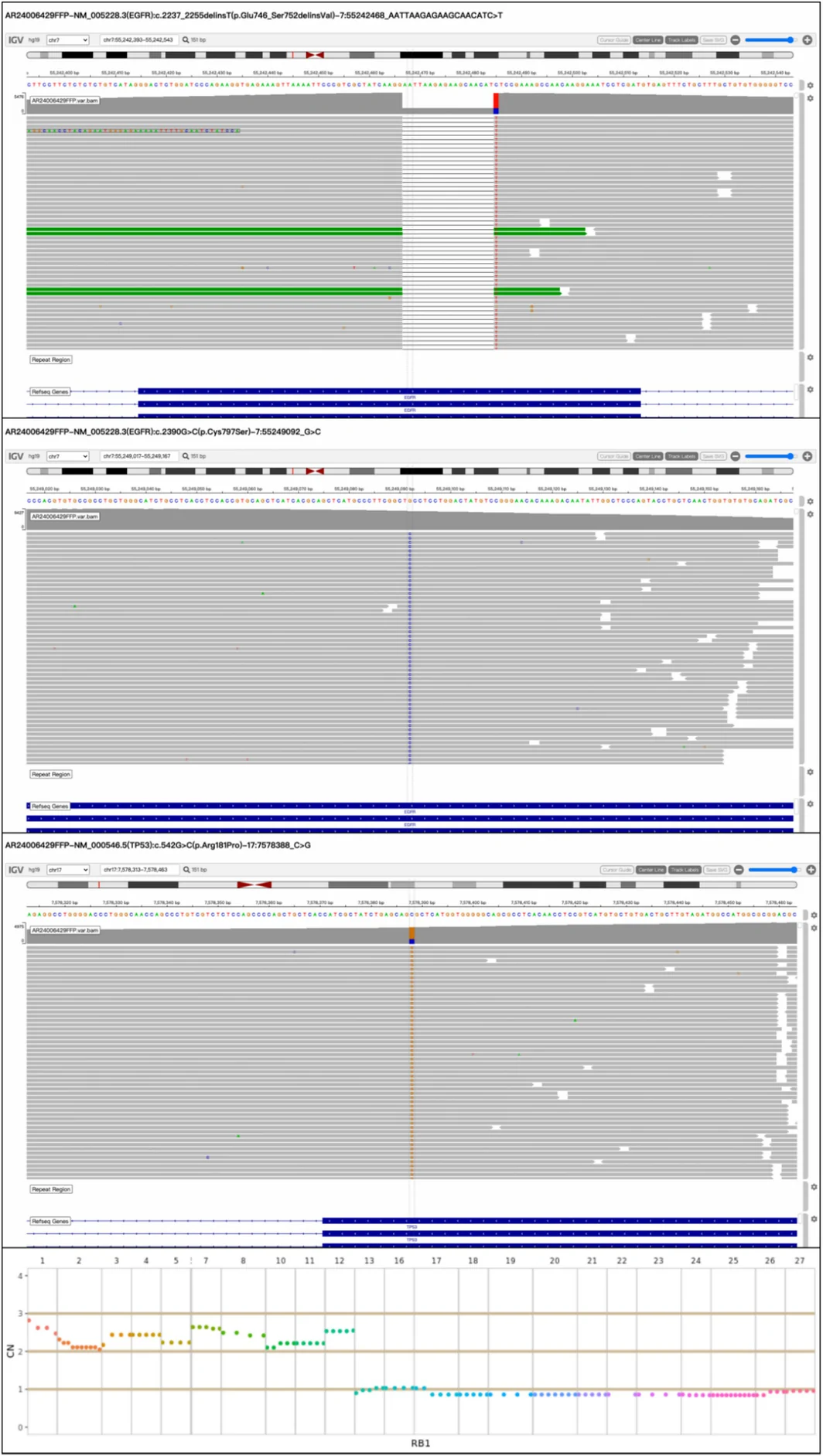
Next‐generation sequencing (NGS) findings of the second biopsy specimen, including EGFR Exon 19 deletion, EGFR Exon 20 C797S mutation, and TP53/RB1 alterations.

**FIGURE 4:**
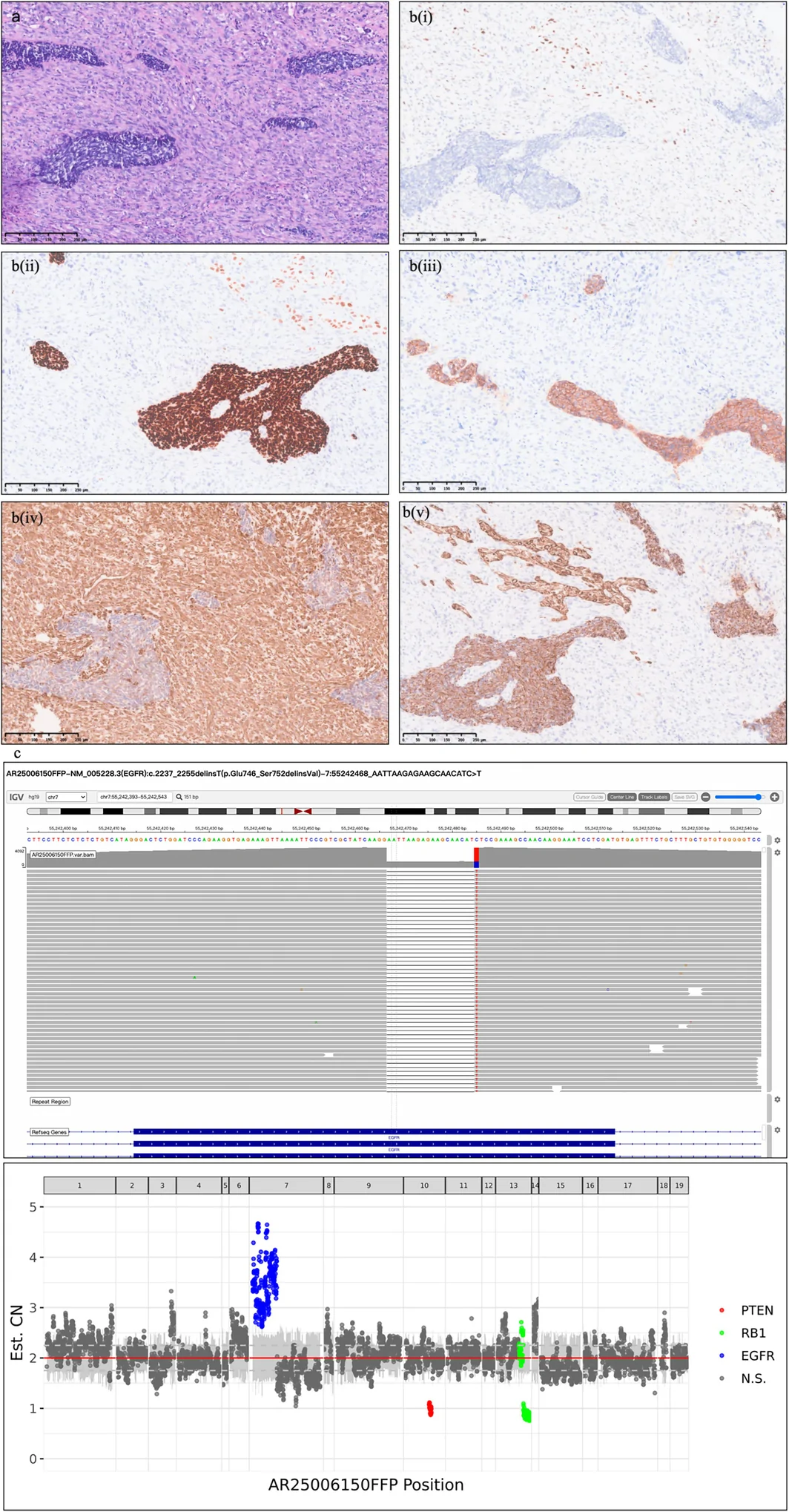
Histological, immunohistochemical, and molecular characteristics of the third biopsy specimen showing sarcomatoid carcinoma with a minor small‐cell neuroendocrine carcinoma component. (a) stained with H&E. (b) IHC stained with (i) thyroid transcription factor‐1 (TTF‐1), (ii) chromogranin A (CgA), (iii) p63, (iv) Vimentin and (v) pan‐cytokeratin (AE1/AE3). (c) NGS indicated (i) EGFR Exon 19 deletion and (ii) PTEN loss.

**FIGURE 5:**
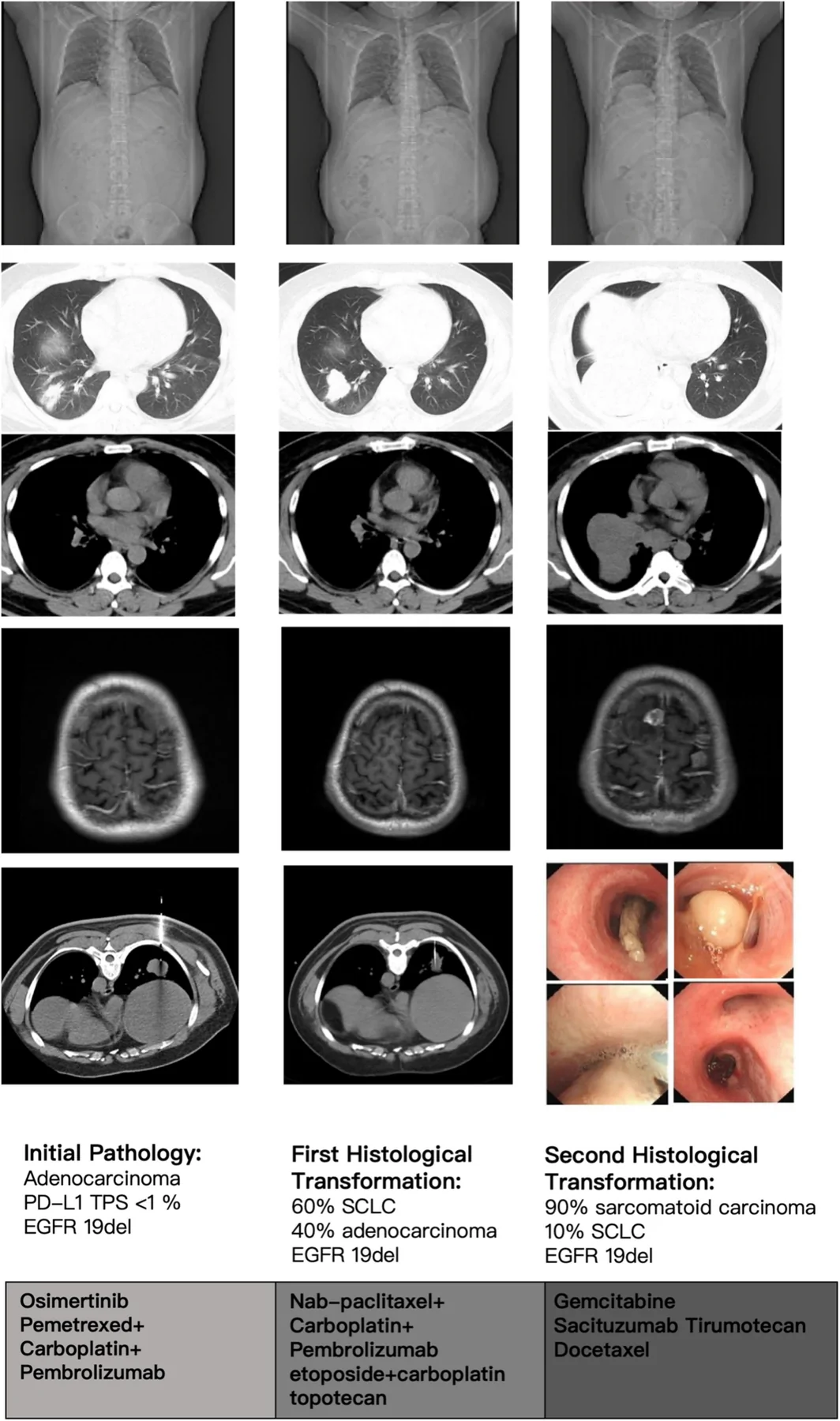
Sequential histological transformations and treatment course of the case.

## 2 Discussion

Histological transformation occurring twice in a patient with EGFR‐mutant lung adenocarcinoma under the selective pressure of systemic therapy is extremely rare. We hereby present a case of sequential histological transformation to underscore its significance and raise clinical awareness. The first histological transformation occurred after 20 months of osimertinib treatment, with the tumor evolving from lung adenocarcinoma to combined small‐cell carcinoma containing both small‐cell lung carcinoma and adenocarcinoma components. NGS revealed that, under the selective pressure of EGFR‐TKI therapy, the tumor evolved through two resistance pathways: one subpopulation of the EGFR‐mutant adenocarcinoma acquired the EGFR C797S resistance mutation [1], while another pre‐existing TP53/RB1‐altered tumor population may have undergone lineage reprogramming toward a neuroendocrine phenotype, contributing to the development of the small‐cell carcinoma [2, 3]. EGFR‐mutant adenocarcinoma with TP53/RB1 co‐inactivation are at a higher risk of transforming to small‐cell carcinoma following EGFR‐TKI resistance [4]. This case illustrated that distinct resistance mechanisms may emerge simultaneously under the selective pressure of EGFR‐TKI therapy [5, 6, 7].

The second histological progression occurred following platinum‐doublet chemotherapy. Rebiopsy revealed a heterogeneous tumor featuring both sarcomatoid carcinoma and small‐cell neuroendocrine carcinoma components. In our case, the sarcomatoid component retained the original EGFR exon 19 deletion and TP53/RB1 alterations, supporting a common clonal origin and further histological evolution rather than the emergence of a de novo tumor. Sarcomatoid transformation of lung adenocarcinoma is relatively rare. This histological transformation is considered to be associated with epithelial‐to‐mesenchymal transition (EMT), which represents one of the mechanisms contributing to resistance under therapeutic pressure from targeted therapies, chemotherapy, and immunotherapy [8, 9, 10]. Concurrently observed PTEN copy number reduction identified by NGS may have contributed to dysregulation of the PI3K/AKT/mTOR pathway and potentially facilitated sarcomatoid transformation together with TP53/RB1 alterations [4, 11]. Consistent with reported data indicating poor prognosis and suboptimal response to chemotherapy in transformed sarcomatoid carcinoma [12, 13], our patient's disease progressed rapidly through multiple lines of therapy. This case highlights the importance of repeated tumor sampling during disease evolution, as histological and molecular reassessment may provide critical information for therapeutic decision‐making in EGFR‐mutant lung cancer with acquired resistance.

## Author Contributions

**Bingyu Huang:** data curation, writing – original draft, formal analysis, investigation. **Tao Lu:** data curation, visualization, formal analysis, investigation. **Ruping Hong:** data curation, formal analysis. **Xiaoyan Liu:** data curation, formal analysis. **Xiaoxing Gao:** data curation, formal analysis. **Yan Xu:** data curation, formal analysis. **Minjiang Chen:** data curation, formal analysis. **Wei Zhong:** data curation, formal analysis. **Mengzhao Wang:** data curation, formal analysis, investigation. **Jing Zhao:** investigation, project administration, writing – review and editing.

## Funding

This study was supported by the Peking Union Medical College Hospital Talent Cultivation Program (Category C, UBJ06503).

## Consent

Informed consent was obtained from the patient. All patient information was de‐identified prior to publication.

## Conflicts of Interest

The authors declare no conflicts of interest.

## Data Availability

The data supporting the findings of this study are available from the corresponding author upon reasonable request.
